# Fatigue and Metabolic Responses during Repeated Sets of Bench Press Exercise to Exhaustion at Different Ranges of Motion

**DOI:** 10.5114/jhk/185524

**Published:** 2024-04-15

**Authors:** Athanasios Tsoukos, Michał Krzysztofik, Michal Wilk, Adam Zajac, Michail G. Panagiotopoulos, Ilias-Iason Psarras, Despina P. Petraki, Gerasimos Terzis, Gregory C. Bogdanis

**Affiliations:** 1 School of Physical Education and Sports Science, National & Kapodistrian University of Athens, Athens, Greece.; 2 Institute of Sport Sciences, Jerzy Kukuczka Academy of Physical Education in Katowice, Katowice, Poland.

**Keywords:** velocity-based training, blood lactate, muscle length

## Abstract

This study compared the acute effects of different ranges of motion (ROM) on fatigue and metabolic responses during repeated sets of bench press exercise. Ten resistance trained men performed three sets to momentary failure with two-min rest intervals at three different ROM: full ROM (FULL), and partial ROM in which the barbell was moved either at the bottom half (BOTTOM) or the top half (TOP) of the full barbell vertical displacement. In TOP, a higher load was lifted, and a higher total number of repetitions was performed compared to FULL and BOTTOM (130 ± 17.6 vs. 102.5 ± 15.9 vs. 98.8 ± 17.5 kg; 55.2 ± 9.8, 32.2 ± 6.5 vs. 49.1 ± 16.5 kg, respectively p < 0.01). Work per repetition was higher in FULL than TOP and BOTTOM (283 ± 43 vs. 205 ± 32 vs. 164 ± 31 J/repetition, p < 0.01). Mean barbell velocity at the start of set 1 was 21.7% and 12.8% higher in FULL compared to TOP and BOTTOM, respectively. The rate of decline in mean barbell velocity was doubled from set 1 to set 3 (p < 0.01) and was higher in FULL than both TOP and BOTTOM (p < 0.001). Also, the rate of mean barbell velocity decline was higher in BOTTOM compared to TOP (p = 0.045). Blood lactate concentration was similarly increased in all ROM (p < 0.001). Training at TOP ROM allowed not only to lift a higher load, but also to perform more repetitions with a lower rate of decline in mean barbell velocity. Despite the lower absolute load and work per repetition, fatigue was higher in BOTTOM than TOP and this may be attributed to differences in muscle length.

## Introduction

Fatigue may be defined as a decrease in the required or expected force and/or power production during repeated muscle actions (Bogdanis, 2012; Enoka and Duchateau, 2008). However, performing repetitions to volitional fatigue during resistance exercise may be desirable if the aim is to induce hypertrophy or increase muscle strength (Lasevicius et al., 2022; Singal et al., 2018), through high metabolic (Goto et al., 2005) or muscular loads (Schoenfeld, 2010) and neural adaptations (Gabriel et al., 2001; Walker et al., 2012). Fatigue may be quantified by measuring the total number of repetitions (Pekünlü and Atalaǧ, 2013), the total work done (Pareja-Blanco et al., 2017), the percentage drop in performance (Tsoukos et al., 2021; Tsoukos and Bogdanis, 2023b) or the slope of performance decrement (Tsoukos and Bogdanis, 2023a).

Muscle length is an important variable influencing fatigue, with exercise at long muscle inducing a greater degree of fatigue (Lee et al., 2007). Muscle length may be manipulated by changing the range of motion (ROM) through which a joint moves during resistance exercise (Haff and Triplett, 2016). In training practice, instead of exercising through full ROM, which is to move the barbell or the external resistance as far as anatomically possible at a given joint, each repetition may be performed through partial ROM, which limits the muscles involved to work at shorter or longer lengths (Newmire and Willoughby, 2020). Previous studies have shown large differences in kinematic, kinetic and surface electromyographic (sEMG) responses, as well as in total training volume when performing resistance exercises at different ROM (Clark et al., 2008; Gillingham et al., 2023; Krzysztofik et al., 2021; Matykiewicz et al., 2023). Furthermore, differences in musculoskeletal mechanics, as well as neural and metabolic responses during exercise at shorter or longer muscle length influence muscle fatigue (Bloomquist et al., 2013; Bogdanis et al., 2019; Maganaris, 2003; Mendonça et al., 2021; Murphy et al., 1996).

When ROM, and thus bar displacement, is large, then mean and peak velocities are higher compared with shorter ROM (Drinkwater et al., 2012; Krzysztofik et al., 2020, 2021, 2023). For example, Krzysztofik et al. (2020, 2021) observed that mean and peak velocities were greater when participants performed repetitions in the bench press exercise using a cambered barbell, which increased bar displacement and ROM compared to the standard straight barbell. Similar findings have been reported for the squat exercise, with higher peak velocity being achieved in full compared with partial ROM against both moderate and high loads (Drinkwater et al., 2012). The number of repetitions is also influenced by ROM, with research showing a negative effect of increased ROM (Krzysztofik et al., 2021, 2023). A slight increase in ROM, and thus muscle length of the muscles involved, by using a cambered barbell during the bench press exercise resulted in a 10% decrease in the number of repetitions performed during three sets (59.1 ± 5 vs. 53.1 ± 5.4 repetitions, *p* < 0.01). Another similar study showed no difference between the two types of barbells (standard vs. cambered) in each of the five performed sets, but overall, participants performed a greater number of repetitions in shorter ROM (49 ± 7 vs. 43 ± 8 repetitions) (Matykiewicz et al., 2023). These inconsistent results may be due to the small displacement differences between the two types of barbells (35 ± 2.3 vs. 41 ± 2.9 cm) (Krzysztofik et al., 2021). However, mechanical loads, rather than simply the number of repetitions should be examined, since this variable is crucial for both fatigue and muscle adaptations (Peterson et al., 2011). Thus, total mechanical load should be quantified and compared between partial and full movements in order to have a clearer picture of the effects of different ROM on load and fatigue during resistance exercise.

It has been reported that during fatiguing protocols to instant exhaustion (e.g., during hypertrophy protocols), blood lactate concentration increases significantly and remains elevated for around 15 min post exercise (Heredia-Elvar et al., 2022; Hernández-Lougedo et al., 2022), while movement velocity of the barbell decreases linearly (Tsoukos et al., 2021) and the number of completed repetitions also decreases during repeated sets of exercise (Heredia-Elvar et al., 2022; Hernández-Lougedo et al., 2022). There is evidence showing that blood lactate responses during resistance exercise depend not only on the total work, but mainly on the frequency of repetitions. Specifically, when time under tension (TUT), and thus total work is equalized, shorter and more frequent repetitions (i.e., 12 repetitions each lasting 3 s vs. 6 repetitions each lasting 6 s) against the same load, result in 20% higher blood lactate concentration and higher sEMG activity (Lacerda et al., 2016). Elevated blood lactate concentration is an indicator of higher glycolytic contribution to energy supply and is related with metabolic disturbances which cause muscle fatigue, such as low levels of pH and phosphocreatine and high inorganic phosphate concentration (Bogdanis et al., 1995; McMahon and Jenkins, 2002). Thus, measuring blood lactate concentration during resistance exercise with different ROM may provide more insight into performance and fatigue levels. This may, in turn, provide information regarding the manipulation of ROM and muscle length during resistance exercise in order to maximize metabolic loads.

Taking into account the possibly large effects of ROM on performance and fatigue during resistance exercise, the prospective use of protocols with different ROM in order to maximize adaptations, and the sparsity of information about distinctively different ROM, we designed a study to examine the effects of the bench press exercise in the first half (BOTTOM) and the second half of ROM (TOP) on movement velocity, fatigue and metabolic responses during repeated sets of exercise. These results were compared with full ROM responses, while care was taken to use the same relative load for each ROM by measuring the respective 1-RM and using a relative load of 65% 1-RM. A moderate load was chosen because it has been shown to have similar sEMG to a heavier load when movement velocity is maximum (Tsoukos et al., 2021). Also, such loads are commonly used during muscle hypertrophy resistance training programs (Schoenfeld et al., 2021) and have also been used to improve muscle power (Rodiles-Guerrero et al., 2022). It was hypothesized that performance would be lower, and fatigue would be higher when exercising at bottom ROM, where muscle length is longer.

## Methods

### Participants

Ten resistance trained men participated in the study (age: 23.2 ± 5.1 years, body height: 1.81 ± 0.07 m, body mass: 81.7 ± 10.1 kg, body fat: 10.3 ± 3.9%). Participants were involved in strength and power sports (gymnastics, soccer, basketball, track & field, handball and volleyball) for at least three years. Inclusion criteria were: (a) resistance training including the bench press exercise for at least three years, and (b) relative strength in the bench press exercise of at least 105% of their body mass (relative 1-RM >1.05 kg•kg^−1^ body mass). Exclusion criteria were: (a) use of nutritional supplements and/or drugs, and (b) musculoskeletal injuries for at least one year prior to the study. After a detailed oral and written explanation of the study protocol, possible risks and the right to cease participation at will, a signed written informed consent form was obtained from each eligible participant. The study was approved by the Ethics Committee of the School of P.E. and Sport Science, National and Kapodistrian University of Athens, Greece (protocol code: 1347; approval date: 24 January 2021) and all procedures were in accordance with the Code of Ethics of the World Medical Association (Helsinki declaration of 1964, as revised in 2013).

### Measures

#### Familiarization

Participants completed three preliminary-familiarization sessions. In the first preliminary visit, anthropometric data were collected and the maximum dynamic bench press strength (1-RM) with FULL ROM was measured. Participants were also familiarized with the experimental procedure by performing three sets of 10 repetitions with 2-min rest intervals, against a load corresponding to 50% of the FULL 1-RM, each one with a different ROM (1^st^ set FULL, 2^nd^ set TOP and 3^rd^ set BOTTOM). In the second and third visits, the 1-RM at the TOP ROM and BOTTOM ROM was evaluated, respectively, and participants repeated the familiarization procedure of the first visit.

#### General and Specific Warm-Up

Before each preliminary and main session, participants completed a standardized warm-up consisting of five minutes of light cycling on a cycle ergometer against 60 W and five minutes of dynamic stretching of the arm and chest muscles (Tsoukos et al., 2019). The general warm-up was followed by a specific one. The specific warm-up performed before the 1-RM assessment included eight repetitions at 50% of the predicted 1-RM, five repetitions at 75% of the predicted 1-RM and three repetitions at 90% of the predicted 1-RM. Before the main trials, the specific warm-up included eight repetitions with 50% of the load that followed (65% of the respective 1-RM) and three minutes later, five repetitions with 75% of the load that followed (Tsoukos et al., 2021).

#### Anthropometric Measurements

Body height was measured to the nearest 0.1 cm with a stadiometer (Charder HM-200P Portstad), and body mass was measured with a scale (TBF-300A Body Composition Analyzer-Tanita). Body fat was estimated from seven skinfold thicknesses (Jackson and Pollock, 1985) measured by a Harpenden skinfold calliper (British Indicators Ltd., Herts, England).

#### Maximum Dynamic Strength (1-RM)

Maximum dynamic strength (1-RM) for the three different ROM in the bench press exercise was assessed on a Smith machine, according to the procedures suggested by the National Strength and Conditioning Association (Baechle et al., 2008). The hips, shoulders and the head were kept in constant contact with the bench during the preliminary and main sessions, and the feet were flat on the floor with a knee angle of approximately 90°. Two experienced spotters (strength & conditioning coaches) ensured safe execution of all tests and verbally encouraged participants. The bar was grasped with a pronated grip slightly wider than shoulder-width (Baechle et al., 2008). The ICC for the 1-RM measurement in our laboratory is 0.92 (Tsoukos and Bogdanis, 2023).

#### Main Trials

During the preliminary and main trials at TOP ROM, two block foam pads wrapped with tapes (total thickness approx. half of the individual full ROM) were placed on the chest of participants to serve as guides of the required ROM. Participants ended each repetition with full elbow extension (180°). The elbow angle was measured by an electro-goniometer (Biopac SS21L, BioPac Systems, Goleta, CA) placed on the upper arm and the forearm (sampling frequency of 2000 Hz). The electro-goniometer was connected to a data acquisition device (MP35, systems Inc., Santa Barbara, CA) and analyses were conducted with the appropriate software (Acqknowledge 4.2.0, Biopac Systems Inc., Santa Barbara, CA). During the preliminary and main trials at BOTTOM ROM, participants were guided by one spotter, who placed their hand near the vertical rail at half distance of the individual FULL ROM from the participants’ chest. Each participant had to touch the bar on the spotter’s hand in every repetition. During all ROM conditions, participants were asked to slightly touch the barbell on their chest or the foam pad (Sakamoto and Sinclair, 2006).

#### Movement Velocity Measurement

A linear position transducer (Tendo Power Analyzer System v. 314, TENDO Sports Machines, Trencin, Slovak Republic) was used to measure movement velocity of the barbell in every repetition of each set. This system has been found to be valid and reliable for the measurement of mean and peak velocity (Garnacho-Castaño et al., 2015). The string of the transducer was set vertically to the barbell, and this was ensured by hanging a small weight from the barbell to the floor before each experimental condition (Salagas et al., 2022).

#### Fatigue Profiles

The fatigue profiles of participants in each set were determined from the rate of decline of peak and mean barbel velocity, calculated as the slope of the linear relationship between velocity and repetitions. The strength of this relationship was evaluated from the coefficient of determination (R2).

#### External Work and Frequency of Repetitions Calculations

External work was calculated as the product of the load under each condition multiplied by the acceleration of gravity (9.81 m·s^−2^) and by the number of repetitions. Total time under tension (TUT) under each condition was calculated from the linear position transducer as the sum of the active duration of all successful repetitions. The active time of a repetition was termed as the time when the barbell was in motion (i.e., during the eccentric and concentric phases of each movement). The frequency of repetitions was calculated by dividing the number of repetitions by TUT.

When exercising with various ROM, each repetition has different duration. In the present study, TUT of the first repetition was longer under FULL (1.1 ± 0.1 s) than TOP and BOTTOM conditions (0.8 ± 0.1 for both, *p* < 0.001). For this reason, performance (mean and peak velocity) values were averaged over a certain number of repetitions at the start of each set (three repetitions for partial ROM, i.e., TOP and BOTTOM, and two repetitions for FULL, respectively), so that the total time under tension at each section (initial and last) was the same for the different ROM. This specific number of repetitions was decided after a pilot study we had conducted which showed that during the execution of a bench press set at maximum intended movement velocity, time under tension of the first three repetitions during TOP or BOTTOM ROM with 65% of 1-RM was equal to the time under tension of the first two repetitions during FULL ROM against an equal relative load (65%-1RM). Time under tension equalization among the different ROM in the present study using the above number of repetitions was confirmed (Table 2). The initial and the last part of each set corresponded to the first and last three repetitions in TOP and BOTTOM ROM, and to the first and last two repetitions in FULL ROM, respectively.

#### Blood Lactate Concentration

Blood lactate concentration was measured from the ear lobe in a capillary blood sample 2 min after the end of the warm-up as well as immediately post (IP) and 3 min (POST3) after the end of the last set of exercise using a portable blood lactate analyzer (Lactate Scout+, EKF Diagnostics, Cardiff, UK).

### Design and Procedures

A repeated measures design was used to compare the acute effects of different ROM on barbell velocity and blood lactate concentration (BL) during three sets to failure with maximum movement velocity in the bench press exercise. Participants completed three preliminary and familiarization sessions, and three counterbalanced and randomized main conditions, 5–7 days apart. Each condition consisted of three sets of the bench press exercise to exhaustion with 2-min rest intervals between sets on a Smith machine performed as fast as possible (movement tempo X:0:X:0), against a load of 65% of 1-RM with three different ROM: (a) full ROM (FULL), (b) partial ROM in which the barbell moved at the bottom half of the full barbell vertical displacement (BOTTOM), and (c) partial ROM in which the barbell moved at the top half of the full barbell vertical displacement (TOP). The dependent variables compared among the three conditions and the three sets were: the 1-RM load, barbell displacement, elbow angle, number of repetitions, mean and peak barbell velocity, total and partial time under tension (TUT) and blood lactate concentration measured at three different time points (pre, immediately after and three minutes after the end of the third set).

### Statistical Analysis

Statistical analyses were conducted using the SPSS Statistics Ver. 23 (IBM Corporation, USA). All data are presented as means and standard deviations (SD). Normality, homogeneity, and sphericity of the data were verified by the Shapiro-Wilk, Levene’s, and Mauchly’s tests. One-way repeated measures analysis of variance (ANOVA) was conducted to detect any differences between the three different ROM in load among conditions. Two-way ANOVA was used to examine changes in the load, the number and frequency of repetitions and blood lactate concentration. Three-way repeated measures analysis of variance (ANOVA) [3 ROM (FULL, TOP, BOTTOM) x 3 sets (1^st^, 2^nd^, 3^rd^) x 2 parts (initial and last)] was conducted to examine differences among the three ROM, sets and parts in barbell velocity and TUT. A Tukey’s post-hoc test was performed when a significant main effect or interaction was observed. The effect sizes for main effects and interactions were determined by Partial eta squared (η2) values; η2 values were classified as small (0.01 to 0.059), moderate (0.06 to 0.137) and large (>0.137). For pairwise comparisons, the effect size (ES) was determined by Hedges’ g (small, <0.3; medium, 0.3–0.8; large, >0.8). Statistical significance was set at *p* < 0.05.

## Results

### Maximum Strength and Load

Maximum strength (1-RM) was 26.8 % and 31.6 % higher in TOP compared with FULL (*p* < 0.001; Hedges' g = 1.6) and BOTTOM (*p* < 0.001; Hedges' g = 1.7, Table 1), respectively. Thus, the load used for the main trials (65% 1-RM) was higher in TOP compared with FULL (*p* < 0.001; Hedges' g = 1.6) and BOTTOM (*p* < 0.001; Hedges' g = 1.7, Table 1).

**Table 1:** Key variables of the bench press exercise at different ROM.

Variable	FULL ROM	BOTTOM ROM	TOP ROM
Maximum strength 1-RM (kg)	102.5 ± 15.9	98.8 ± 17.5	130.0 ± 17.6^*^
Relative strength (kg•kg^−1^ body mass)	1.26 ± 0.13	1.21 ± 0.15	1.60 ± 0.19^*^
65% 1-RM (kg)	67.3 ± 10.4	65.0 ± 11.2	84.5 ± 10.5^*^
Elbow starting angle (^o^)	78 ± 12	77 ± 9	114 ± 4^*^
Elbow ending angle (^o^)	179 ± 2	128 ± 9^‡^	180 ± 1
Barbell vertical displacement (cm)	42.7 ± 3.4	25.5 ± 2.3**^#^**	24.5 ± 2.3**^#^**
Total number of repetitions	32.2 ± 6.5	49.1 ± 16.5^#^	55.2 ± 9.8^#^
Number of Repetitions in set 1	16.0 ± 2.3	23.4 ± 5.6^#^	28.4 ± 4.9^*^
Number of Repetitions in set 2	9.9 ± 2.5^†^	15.4 ± 6.3^#†^	16.0 ± 2.7^#†^
Number of Repetitions in set 3	6.3 ± 3.0^§^	10.3 ± 5.9^#§^	10.8 ± 4.7^#§^
Total external work (J)	9182 ± 2723	7959 ± 2654	11512 ± 3674^*^
Average work per repetition (J/rep)	283 ± 43	164 ± 31^#^	205 ± 32^*^
Total active TUT (s)	49.6 ± 7.5	46.0 ± 10.1	57.1 ± 9.6^*^
TUT initial repetitions SET 1 (s)	2.2 ± 0.2	2.3 ± 0.4	2.4 ± 0.2
Total inactivity time (s)	3.4 ± 1.4	3.2 ± 1.1	3.2 ± 1.7
Average frequency of repetitions (Hz)	0.61 ± 0.09	0.99 ± 0.19**^#^**	0.92 ± 0.12**^#^**

Elbow extension = 180°; TUT: time under tension; *: p < 0.001 from FULL and BOTTOM; #: p < 0.001 from FULL; ‡: from FULL and TOP. †: p < 0.01 from set 1 in the corresponding ROM; §: p < 0.01 from set 2 in the corresponding ROM.

### Vertical Displacement of the Barbell

The two-way ANOVA (ROM × SET) did not show a significant interaction for the vertical displacement of the barbell (*p* = 0.65; η^2^ = 0.06). However, there were significant main effects for ROM (*p* < 0.001; η^2^ = 0.97; Table 1) and SET (*p* < 0.01; η^2^ = 0.49). Tukey post hoc tests showed that the vertical displacement of the barbell was greater in FULL ROM compared to TOP (*p* < 0.001; Hedges' g = 5.7) and BOTTOM (*p* < 0.001; Hedges' g = 6.0), with no significant difference between TOP and BOTTOM (*p* > 0.05, Table 1). Also, barbell displacement was significantly lower during set 3 compared to set 1 by 1.3 ± 1.3 cm (SET 1: 31.5 ± 0.09 cm vs. SET 3: 30.3 ± 0.09 cm; *p* < 0.01).

### Number of Repetitions and External Work Done

The two-way ANOVA (ROM × SET) showed a significant interaction (*p* < 0.001; η^2^ = 0.66) for the number of repetitions. Tukey post hoc tests revealed that the number of repetitions progressively decreased from set 1 to set 3 under all conditions (*p* < 0.001, Figure 2). A significantly higher number of repetitions was performed under TOP compared to FULL and BOTTOM conditions only in set 1 (Figure 3), while in sets 2 and 3 the number of repetitions was higher under TOP and BOTTOM compared to FULL ROM condition, with no difference between TOP and BOTTOM. The latter was also true for the total number of repetitions which was 71.4 % and 63.4 % higher under FULL compared to TOP and BOTTOM conditions, with no difference between them (TOP from FULL: *p* < 0.01; Hedges' g = 2.6; BOTTOM from FULL: *p* < 0.01; Hedges' g = 1.3, BOTTOM from TOP: *p* = 0.29, Hedges' g = 0.43, Table 1). External work per repetition in FULL was 42% higher compared with TOP and 27% higher compared with BOTTOM (all *p* < 0.001, Table 1). Also, work per repetition was higher in TOP than BOTTOM (*p* < 0.001, Table 1). However, total external work was 20.2% and 30.9% lower in FULL (*p* < 0.01; Hedges' g = 0.69) and BOTTOM (*p* < 0.01; Hedges' g = 1.06) compared with TOP, with no difference between FULL and BOTTOM conditions (Table 1).

**Figure 1:**
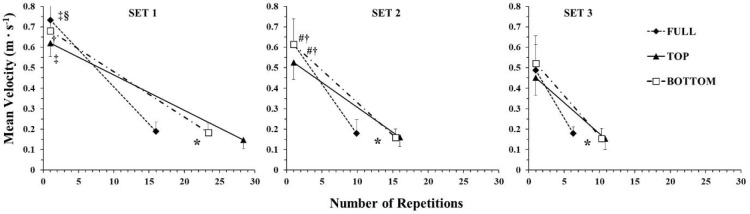
Mean barbell velocity during the different ROM conditions in the three sets. *: *p <* 0.01 from the initial repetitions under all ROM conditions; ‡: *p <* 0.01 from the initial repetitions of the 2^nd^ and the 3^rd^ set in the corresponding ROM; †: *p <* 0.01 from the initial repetitions of the 3^rd^ set in the corresponding ROM; §: *p <* 0.01 from TOP and BOTTOM ROM in the corresponding SET and repetitions; #: *p <* 0.01 from TOP ROM in the corresponding SET and repetitions

**Figure 2:**
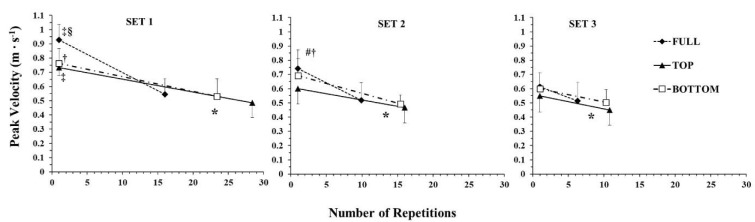
Peak barbell velocity during the different ROM conditions in the three sets. *: *p <* 0.01 from the initial repetitions under all ROM conditions; ‡: *p <* 0.01 from the initial repetitions of the 2^nd^ and the 3^rd^ set in the corresponding ROM; †: *p <* 0.01 from the initial repetitions of the 3^rd^ set in the corresponding ROM; §: *p <* 0.01 from the TOP and BOTTOM ROM in the corresponding SET and repetitions; #: *p <* 0.01 from the TOP ROM in the corresponding SET and repetitions

**Figure 3:**
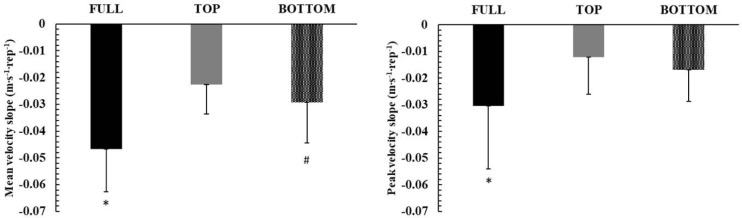
The rate of decline (slope) during the three different ROM conditions, irrespective of the set (all sets considered together). *: *p* < 0.01 from TOP and BOTTOM ROM; #: *p* < 0.05 from TOP ROM

Also, total active TUT was higher in TOP compared with FULL (*p* = 0.0497; Hedges' g = 0.8) and BOTTOM (*p* = 0.0036; Hedges' g = 1.1). Frequency of repetitions was higher in TOP and BOTTOM compared with FULL (TOP from FULL: *p* < 0.01; Hedges' g = 2.88; BOTTOM from FULL: *p* < 0.01; Hedges' g = 2.48, Table 1).

### Equalization of Partial Time under Tension (TUT) between Different ROM

ANOVA showed no three-way (ROM × SET × PART) or two-way interactions for condition (i.e., ROM, *p* > 0.05; η^2^ = 0.10). Thus, the time for the initial and last repetitions was equalized across the three conditions, allowing valid comparisons. A two-way interaction was found between SET and PART (*p* = 0.048; η^2^ = 0.29). Tukey post hoc tests indicated that, irrespective of the set and condition, TUT in the last repetitions (s) was almost 2-fold longer compared to the initial repetitions (4.8 ± 1.2 vs. 2.5 ± 0.5 s, *p* = 0.001).

### Mean and Peak Barbell Velocity

The time course of changes in the mean barbell velocity is presented in Figure 1. ANOVA showed a three-way interaction (*p* < 0.01; η^2^ = 0.35) for mean velocity. Tukey post-hoc tests revealed that mean barbell velocity in the first repetitions (i.e., initial part) was always higher than in the final repetitions (i.e., last part), and the initial mean barbell velocity decreased as sets were repeated, which shows that fatigue was observed under all conditions and in all sets. Mean barbell velocity at the end of each set was similar under all conditions. However, mean barbell velocity in the first repetitions of set 1 was 21.7% and 12.8% higher in FULL compared to TOP and BOTTOM, respectively. In set 2, mean barbell velocity in the initial part in FULL was similar to BOTTOM, and mean barbell velocity in TOP was lower than in both FULL and BOTTOM. In set 3, mean barbell velocity in the initial part was similar under all conditions (Figure 1). The minimum barbell velocity was observed in the last repetition and was similar under all conditions and in all sets (range from 0.15 ± 0.04 to 0.19 ± 0.04 m·s^−1^).

The time course of changes in the peak barbell velocity is presented in Figure 2. ANOVA showed a three-way interaction (*p* = 0.019; η^2^ = 0.27) in peak barbell velocity. Tukey post-hoc tests revealed that peak barbell velocity in the first repetitions (i.e., initial part) was always higher than in the final repetitions (i.e., last part), and the initial peak velocity decreased as sets were repeated, which shows that fatigue was evident under all conditions and in all sets. Peak barbell velocity at the end of each set was similar under all conditions. However, peak barbell velocity in the first repetitions of set 1 was 26.5% and 21.6% higher in FULL compared to TOP and BOTTOM, respectively. In set 2, peak barbell velocity in the initial part in FULL was similar to BOTTOM and higher than TOP. In set 3, peak barbell velocity in the initial part was similar under all conditions (Figure 1).

### Changes in Fatigue Profiles during Exercise

The rates of mean and peak velocity drop are presented in Figure 3. The drop in mean and peak velocity across repetitions in each set was linear (r = 0.80–0.99, *p* < 0.001). Two-way ANOVA (ROM x SET) did not show an interaction for both mean and peak velocity slopes (mean velocity slope: *p* = 0.16; η^2^ = 0.16, peak velocity slope: *p* = 0.81; η^2^ = 0.04). However, there was a main effect for ROM in both mean and peak barbell velocity (*p* < 0.0001; η^2^ = 0.84, and *p* < 0.01; η^2^ = 0.58, respectively). Tukey post-hoc tests showed that there was a greater decline in mean and peak velocity in FULL compared with TO *P* (*p* < 0.001; Hedges' g = 1.67 and g = 0.90, respectively) and BOTTOM (*p* < 0.001; Hedges' g = 1.07 and g = 0.69, respectively, Figure 3). Also, the rate of mean barbell velocity decline was higher in BOTTOM compared to TOP (*p* = 0.045; Hedges' g = 0.49). No differences were observed between TOP and BOTTOM in the rate of peak barbell velocity decline (*p* = 0.44).

**Figure 4:**
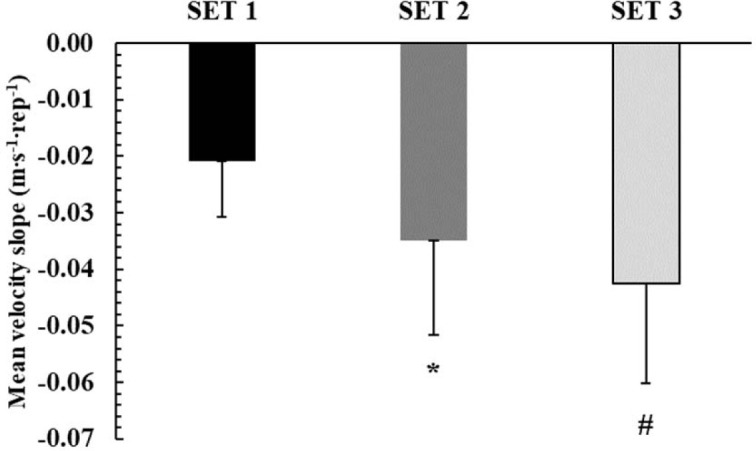
The rate of decline in mean barbell velocity (slope) during the three sets. *: *p* < 0.01 from SET 1; #: *p* < 0.05 from SET 1 and SET 2

The rate of decline in mean barbell velocity increased in each repeated set (*p* < 0.0001; η^2^ = 0.83). Tukey post-hoc tests showed that the rate of the mean barbell velocity decline increased progressively and was doubled from set 1 to set 3 (−0.021 ± 0.01 vs. −0.043 ± 0.02 m·s^−1^·rep^−1^; *p* < 0.01; Figure 4). No set effect observed for the rate of decline in peak barbell velocity (*p* = 0.58).

### Blood Lactate Concentration

**Figure 5:**
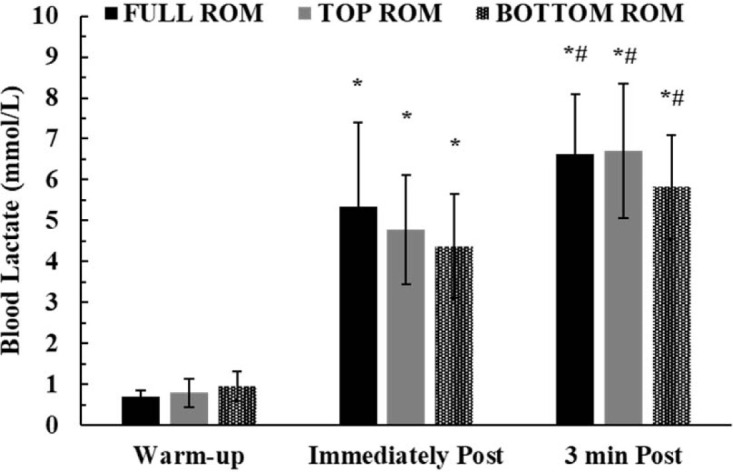
Blood lactate concentration under the three experimental conditions. *: *p* < 0.01 from the warm-up; #: *p* < 0.01 from immediately post exercise

Two-way ANOVA did not show a significant interaction for blood lactate concentration (*p* = 0.07; η^2^ = 0.21). However, there was a significant main effect of time (*p* < 0.0001; η^2^ = 0.92). Tukey’s post hoc tests showed that blood lactate concentration progressively increased from pre to immediately post and 3 min after the end of all protocols under all conditions (*p* < 0.01; Figure 5).

## Discussion

This study compared performance and fatigue during repeated sets of the bench press exercise at three different ranges of motion (FULL, TOP and BOTTOM). The main findings were that when exercising at TOP ROM, participants not only lifted a higher load, but also performed more external work and had longer TUT compared to FULL and BOTTOM conditions. As seen from the rate of decline of peak and mean barbell velocity and the number of repetitions performed, fatigue was higher in FULL than both TOP and BOTTOM. Mean and peak barbell velocities were higher in the initial repetitions in FULL compared to TOP and BOTTOM, but as the number of sets progressed mean and peak barbell velocities decreased, and were equalized under all conditions, with TOP demonstrating the lowest value of all conditions at the initial part of set 2.

One main finding of the present study was the greater fatigue observed in TOP compared with the other two ROM. This was demonstrated as a greater rate of decline in peak and mean barbell velocity (Figures 1 and 2), and consequently, a higher slope of the velocity vs. repetitions relationship (Figure 3). Also, the number of repetitions per set was significantly lower in FULL compared with TOP and BOTTOM (Table 1), showing the inability of participants to maintain the required force and power production (Bogdanis, 2012; Enoka and Duchateau, 2008). One possible explanation for the greater fatigue in FULL may be the 27–42% higher external work per repetition compared to TOP and BOTTOM (Table 1). In FULL, participants had to move the barbell for a longer distance, which increased external work done and prolonged the propulsive (and eccentric) phase of the movement. This made each repetition more demanding in terms of energy expenditure and contributed to the higher rate of barbell velocity decline and fatigue (Scott et al., 2011). Another factor contributing to muscle fatigue may be the velocity of movement (Tsoukos et al., 2021; Tsoukos and Bogdanis, 2023b). Specifically, moving at higher velocities not only made exercise more energy demanding, but also recruited more fast-twitch motor units, which are less fatigue resistant than slower ones (Del Vecchio et al., 2019; Thorstensson and Karlsson, 1976). The faster movement when exercising at FULL than at partial ROM has been previously reported in the literature (Drinkwater et al., 2012; Krzysztofik et al., 2020, 2021), and this is explained by the longer propulsive phase, which is coupled with a longer acceleration phase, resulting in higher peak and mean velocity compared with any shorter ROM partial repetition. Notably, the propulsive phase constitutes 93% of the concentric duration in the bench press exercise at a similar load as that used in the present study, i.e., 65% of 1-RM (Sanchez-Medina et al., 2010). Thus, the ~20% higher mean and peak barbell velocity in FULL along with the higher work done per repetition compared with the partial ROM conditions may have contributed to the greater rate of fatigue and the lower number of repetitions.

Another interesting finding of the present study was that the rate of decline in mean barbell velocity was greater in BOTTOM compared to TOP (Figure 3). This finding suggests that fatigue is greater at all sets when exercising at the lower compared to the higher part of the bench press exercise full ROM (Figure 3), although the 11% difference (49 vs. 55 repetitions, Table 1) between BOTTOM and TOP in the number of repetitions was not statistically significant but had a moderate effect size (*p* = 0.29, Hedges' g = 0.43). The greater rate of performance decline may be partially explained by the effects of muscle length on performance and fatigue. When performing the bench press exercise at the lower part of the full ROM (BOTTOM), all the main agonist muscles (pectoralis major, anterior deltoid and triceps brachii) operate at a longer muscle length compared to exercise at the higher part of ROM (TOP). Previous studies have shown that fatigue is affected by muscle length, with fatiguability increasing at longer muscle length or wider angles compared to shorter muscle length (Lee et al., 2007; Tsoukos et al., 2016). Interestingly, the load was 31.6% lower under the BOTTOM compared to the TOP condition, due to the differences in 1-RM. Therefore, despite the lower absolute load and work per repetition (Table 1), fatigue was higher in BOTTOM than TOP, and this may be attributed to the differences in muscle length. Previous studies have shown that fatigue is greater at longer muscle lengths, and this is attributed to both metabolic and neural factors, such as a decreased blood flow and modifications in muscle activation (Lee et al., 2007; Rassier, 2000).

The fact that 1-RM was more than 30% higher in TOP than the other two ROM is a combination of the shorter-closer to optimal-muscle length and more favorable musculoskeletal mechanics as the arms operate at a wider elbow angle and the resistive torque at the elbow and shoulder is lower (Bloomquist et al., 2013; Bogdanis et al., 2019; Murphy et al., 1996). Previous studies confirm the magnitude of strength differences among different ROM in the bench press exercise. For example, one study reported that the maximal isometric force was almost 30% higher at an elbow angle of 120° compared to an angle of 90° in the bench press exercise (Murphy et al., 1996). Interestingly, the elbow starting angles at BOTTOM and TOP ROM were close to these values, resulting in a similar force and strength difference (TOP: 114 ± 4° and BOTTOM: 77 ± 9°; Table 1). Thus, it may be argued that TOP ROM may be used to increase absolute loading in the bench press exercise, without increasing fatigue. Despite the much higher load, fatigue in TOP was lower compared to both BOTTOM and FULL, as shown by the lower slope of the velocity vs. repetitions relationship, the total number of repetitions and total external work performed (Table 1). The adjustment of the load according to the ROM-specific 1-RM is an advantage of the present study, as it allows to demonstrate the lower fatigability and higher performance when exercising at TOP ROM. Although there are some suggestions that adaptation to resistance training such as muscle hypertrophy may be higher when exercising at long muscle length, more work is needed to explore the benefits and adaptation to training at shorter muscle length in multi-joint exercises such as the bench press and the squat, especially when adjusting the load according to ROM-specific strength.

As seen in Figures 1 and 2, mean and peak barbell velocity at the end of all sets and under all conditions was similar. This is a typical observation when performing repetitions to failure (Tsoukos et al., 2021; Tsoukos and Bogdanis, 2023b) and is related to the “minimum velocity threshold”, which is the slowest velocity at which a repetition can be completed for a given exercise (García-Ramos, 2023; Weakley et al., 2021). The values measured in the present study, i.e., from 0.15 ± 0.04 to 0.19 ± 0.04 m·s^−1^, are in agreement with the literature (Pérez-Castilla et al., 2021; Weakley et al., 2021) and denote that in this exercise participants reach a relatively slow barbell velocity at exhaustion. The decline in barbell velocity as repetitions are performed as fast as possible depends on the velocities at the start and the end of the set, as well as on the number of repetitions performed. Thus, if the endpoint of barbell velocity is the same across conditions and sets, and the number of repetitions in sets 2 and 3 the same in TOP and BOTTOM, then the starting velocity may be an important factor for fatigue. Moving slower may positively modify metabolic responses and muscle motor unit contribution in relation to fatigue (Martins-Costa et al., 2016; Taylor et al., 2016). Since a 30% higher load was used in TOP, it is difficult to separate out the contribution of the heavier load in reducing the ability of participants to exercise at high velocities at the start of each set. In any case, differences between conditions in barbell velocity disappeared in set 3, as the peak and mean barbell velocities in the first repetitions were reduced considerably compared to previous sets (Figures 1 and 2).

Blood lactate concentration is an indirect index of glycolytic contribution to energy supply, albeit without offering any insights into muscle metabolism (Theofilidis et al., 2018). Blood lactate concentration increased similarly to around 4 mmol/L under all conditions immediately after the end of the third set, which increased to about 6.5 mmol/L 3 min later in accordance with previous research (Chatel et al., 2016). Blood lactate concentration after high intensity exercise of this volume is expected to reach a peak after 1.3 to 3.3 min from the cessation of exercise (Chatel et al., 2016), but information on this is sparse. The level of blood lactate concentration reached after this protocol is in line with previous studies reporting comparable values after 3 sets to failure using a relative load corresponding to 70% of 1-RM (Heredia-Elvar et al., 2022; Hernández-Lougedo et al., 2022). Despite the relatively small muscle mass, the considerable increase in blood lactate concentration within only three sets of the bench press exercise may indicate high metabolic stress and considerable glycolytic contribution (de Freitas et al., 2017; Schoenfeld, 2013). However, the causes of fatigue in this type of exercise do not only depend on metabolic acidosis, but also on the depletion and the rate of replenishment of phosphocreatine, with the concomitant changes in inorganic phosphate accumulation (Bogdanis et al., 1995; McMahon and Jenkins, 2002). The relatively short (2 min) recovery interval may not allow complete phosphocreatine resynthesis, which is important for high power generation (Bogdanis et al., 1995, 1996) especially when bouts of exercise are repeated (Dawson et al., 1997). This was demonstrated in the present study by the similar slope of peak velocity decline in TOP and BOTTOM (Figure 3), which may indicate the importance of phosphocreatine for performance at the start of each effort (Bogdanis et al., 1995). Moreover, glycolysis, and thus lactate production, is severely reduced or even blocked when bouts of intense exercise of short duration are repeated (Gaitanos et al., 1993). This, coupled with the complex nature of lactate kinetics and turnover, makes blood lactate concentration an inadequate method to provide considerable insight into fatigue during this type of intense exercise. Performing bench press repetitions as fast as possible results in pronounced fatigue from set to set, as shown by the ~40% decrease in the number of repetitions in the second compared to the first set under all conditions, followed by another 35% decrease in the number of repetitions from set 2 to set 3. As noted above, fatigue was evident not only by the drop in the number of repetitions, but also by the decline in barbell velocity which decreased sharply in every set (Figure 4).

Although performance and fatigue considerably differed among the three ROM, as reflected by several performance indicators such as the slope of barbell velocity decline, the number and frequency of repetitions and total external work, blood lactate concentration was similar under all conditions and was therefore unrelated to all the above variables. This was also the case for TUT, which has been put forward as an important factor for inducing high metabolic stress, although this has also been disputed (Gentil et al., 2006). Movement tempo, especially during the eccentric phase, has been suggested to largely affect metabolic responses during resistance exercise (Wilk et al., 2018). Wilk et al. (2018) noted that a slower eccentric phase may augment metabolic responses to resistance exercise, despite the lower number of repetitions to exhaustion. However, in the present study, movement tempo was as fast as possible (X:0:X:0) under all conditions and this may partially explain the similar blood lactate concentrations. This points out that further research is needed to quantify loading not only by taking into account loads, repetitions and bar displacement, but by measuring forces and joint torque, especially in the protocol used in the present study where the execution of movement was as fast as possible, involving high accelerations and thus forces applied on the barbell.

## Conclusions

The present study showed that training at TOP ROM in the bench press exercise allows participants not only to lift a higher load, but also to perform more external work with a lower rate of decline in mean barbell velocity compared to FULL and BOTTOM. During the initial repetitions of the 1^st^ and 2^nd^ sets the mean and peak velocities were higher during FULL compared to partial ROM and velocities decreased to an equal value in the final repetitions. Blood lactate concentration was equal among different ROM indicating considerable contribution of glycolysis to this type of a maximum intended velocity resistance protocol. However, blood lactate concentration could not explain the different performance and fatigue profiles among different ROM. These findings may be applied in training practices in the bench press and other similar multi-joint exercises. Further research is needed to show the effectiveness and differences between TOP and BOTTOM ROM, with equated relative loads in muscle hypertrophy, strength and power. Moreover, quantification of loads by measuring forces applied, rather than loads on the bar, is necessary in this protocol of exercise where high accelerations and thus force fluctuations are seen due to the maximum intended velocity execution. sEMG data may also offer more insight into the causes of fatigue in these different ROM protocols.
